# Rainbow Kaposi's Sarcoma-Associated Herpesvirus Revealed Heterogenic Replication with Dynamic Gene Expression

**DOI:** 10.1128/JVI.01565-19

**Published:** 2020-03-31

**Authors:** Ken-ichi Nakajima, Sara Guevara-Plunkett, Frank Chuang, Kang-Hsin Wang, Yuanzhi Lyu, Ashish Kumar, Guillaume Luxardi, Chie Izumiya, Athena Soulika, Mel Campbell, Yoshihiro Izumiya

**Affiliations:** a Department of Dermatology, School of Medicine, University of California Davis, Sacramento, California, USA; b Department of Biochemistry and Molecular Medicine, School of Medicine, University of California Davis, Sacramento, California, USA; c Viral Oncology and Pathogens-Associated Malignancies Initiative, University of California Davis Comprehensive Cancer Center, Sacramento, California, USA; University of Southern California

**Keywords:** KSHV, transcription, single cells, Kaposi's sarcoma-associated herpesvirus, LANA, ORF6, reactivation, gene expression, herpesviruses, heterologous gene expression

## Abstract

Molecular mechanisms of Kaposi’s sarcoma-associated herpesvirus (KSHV) reactivation have been studied primarily by measuring the total or average activity of an infected cell population, which often consists of a mixture of both nonresponding and reactivating cells that in turn contain KSHVs at various stages of replication. Studies on KSHV gene regulation at the individual cell level would allow us to better understand the basis for this heterogeneity, and new preventive measures could be developed based on findings from nonresponding cells exposed to reactivation stimuli. Here, we generated a recombinant reporter virus, which we named “Rainbow-KSHV,” that encodes three fluorescence-tagged KSHV proteins (mBFP2-ORF6, mCardinal-ORF52, and mCherry-LANA). Rainbow-KSHV replicated similarly to a prototype reporter-KSHV, KSHVr.219, and wild-type BAC16 virus. Live imaging revealed unsynchronized initiation of reactivation and KSHV replication with diverse kinetics between individual cells. Cell fractionation revealed temporal gene regulation, in which early lytic gene expression was terminated in late protein-expressing cells. Finally, isolation of fluorescence-positive cells from nonresponders increased dynamic ranges of downstream experiments 10-fold. Thus, this study demonstrates a tool to examine heterogenic responses of KSHV reactivation for a deeper understanding of KSHV replication.

**IMPORTANCE** Sensitivity and resolution of molecular analysis are often compromised by the use of techniques that measure the ensemble average of large cell populations. Having a research tool to nondestructively identify the KSHV replication stage in an infected cell would not only allow us to effectively isolate cells of interest from cell populations but also enable more precise sample selection for advanced single-cell analysis. We prepared a recombinant KSHV that can report on its replication stage in host cells by differential fluorescence emission. Consistent with previous host gene expression studies, our experiments reveal the highly heterogenic nature of KSHV replication/gene expression at individual cell levels. The utilization of a newly developed reporter-KSHV and initial characterization of KSHV replication in single cells are presented.

## Abstract (precis)

Sensitivity and resolution of molecular analysis are often compromised by the use of techniques that measure the ensemble average of large cell populations. Having a research tool to nondestructively identify the KSHV replication stage in an infected cell would not only allow us to effectively isolate cells of interest from cell populations but also enable more precise sample selection for advanced single-cell analysis. We prepared a recombinant KSHV that can report on its replication stage in host cells by differential fluorescence emission. Consistent with previous host gene expression studies, our experiments reveal the highly heterogenic nature of KSHV replication/gene expression at individual cell levels. The utilization of a newly developed reporter-KSHV and initial characterization of KSHV replication in single cells are presented.

## INTRODUCTION

Molecular and genetic heterogeneity, even among cells of a single tissue type or clonal line, is increasingly evident with the use of new single-cell technologies (1–3). Next-generation sequencing (NGS) enables researchers to routinely obtain millions of sequence reads that reveal genomic mutations, transcriptome variations, and epigenetic differences at the single-cell level (3–5). New developments in mass cytometry have also enabled analysis of cell surface protein expression and metabolites at single-cell resolution (6–9). Thus, advancing single-cell technologies are revolutionizing experimental approaches and allowing researchers to investigate cell-to-cell heterogeneity with unprecedented depth and understand biological outcomes as a sum of individual effects.

Heterogenic cellular responses are especially profound when we consider herpesvirus reactivation from latently infected cells as one of the biological events. Kaposi’s sarcoma-associated herpesvirus (KSHV), also designated human herpesvirus 8, is one of eight human herpesviruses and was discovered in 1994 (10). Since then, KSHV has been identified as the causative agent of Kaposi’s sarcoma (10–12) and two human lymphoproliferative diseases, primary effusion lymphoma (PEL) and AIDS-related multicentric Castleman’s disease (13–16). In those cancer cells, KSHV establishes latent infection in which all but a few viral genes are silenced. External stimuli, such as inhibition of histone deacetylase, expression of strong viral transactivator (ORF50/K-Rta), and/or incubation with 12-*O*-tetradecanoylphorbol 13-acetate (TPA), trigger KSHV reactivation from latently infected cells (17, 18). As with immune cell response to antigens, cell-to-cell variation of KSHV reactivation has been documented (19, 20), along with heterogeneity of response among viral episomes in an individual host cell nucleus (21). Therefore, a higher resolution of analyses is important to identify underlining molecular mechanisms of such heterogenous responses. Since all viral genomes in an infected cell are essentially identical, differences in response to stimulation likely are mediated by host factors; this also makes studies on KSHV gene expression from the “designable” viral minichromosome a unique tool to probe differences in the cellular environment (21, 22).

Like other herpesviruses, KSHV gene transcription occurs in a cascade fashion and is initiated by the expression of a strong transactivator, K-Rta (KSHV replication and transactivation) (23, 24). K-Rta either directly or indirectly binds to viral promoters. By doing so, K-Rta can activate at least 33 viral promoters in an isolated reporter study (25). Those target genes include DNA replication-related genes (e.g., ORF6, ORF59, and K8) and all of the KSHV long noncoding RNAs (PAN RNA, T1.5, and T0.7). Among the genes activated in the early lytic replication phase, ORF6 is known as an early gene and participates in viral DNA replication (26). KSHV ORF6 protein is a homolog of the herpes simplex virus 1 (HSV-1) ICP8 and Epstein-Barr virus (EBV) BALF2 proteins. Similar to those homologous proteins, KSHV ORF6 protein can bind to single-stranded DNA and is essential for lytic DNA replication by forming a replication compartment (RC) (27). After the onset of early gene expression, late genes that usually encode viral structural proteins are expressed. Five viral proteins, ORF18, -30, -31, -34, and -66, were reported to form a viral preinitiation complex (vPIC) for late gene transcription in the presence of the newly replicating DNA (28–30). The expression of many KSHV structural proteins, including ORF52 protein, then was transcribed by cellular RNA polymerase II.

On the other hand, a few proteins, including the latency-associated nuclear antigen (LANA), are abundantly expressed in the nucleus during latent infection (31). LANA is a multifunctional protein involved in the recruitment of cellular machineries for viral DNA replication and segregation of the replicated viral DNAs to daughter cells through interaction with cellular DNA replication factors, chromatin-modifying enzymes, and mitotic apparatus (32–34). LANA directly binds to the terminal repeat (TR) region of the viral genome and associates with nucleosomal proteins to tether to the host cell chromatin (35, 36). Tight binding to the 35 copies of the TR sequences concentrates LANA proteins on the KSHV genome in the nucleus of infected cells, which makes them visible as “LANA dots” under fluorescence microscopy (37). Accordingly, a LANA dot represents a single viral episome in the nucleus; therefore, the fluorescence signal can be used to measure the viral DNA copy number in an infected cell nucleus (38).

In this study, we constructed a new reporter-KSHV that employs three different fluorescent viral proteins to indicate the stage of KSHV lytic replication in individual host cells. Using this construct, we found that KSHV replication and transcription are indeed highly heterogeneous processes. By sorting reactivating cells from nonresponder cells in a culture dish, we improved the precision of transcription analyses to reveal up- and downregulation of early gene expression in a replication stage-dependent fashion from the same culture dish at any given time point.

## RESULTS

### Preparation of Rainbow-KSHV with BAC16.

**FIG 1:**
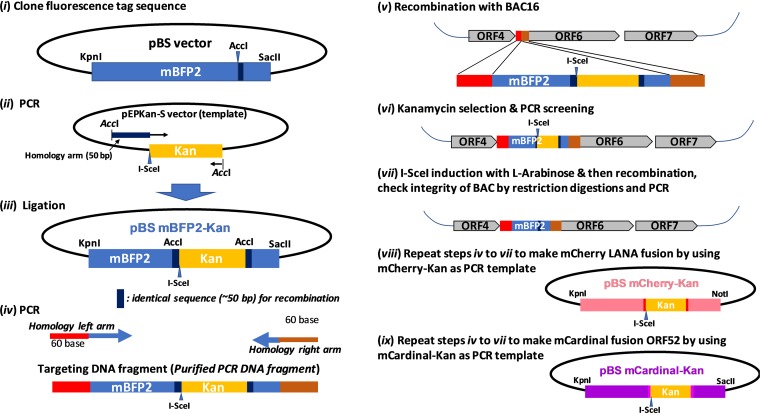
Schematic diagram of procedures to prepare Rainbow-KSHV. (i) The cDNA of fluorescence tag coding sequence (mBFP2, mCardinal, or mCherry) first was cloned into pBS vector. (ii and iii) The kanamycin cassette with I-SceI recognition sequence along with 50-bp homology sequence was amplified by PCR and cloned into the indicated unique restriction enzyme site (AccI or PstI). (iv and v) The resulting plasmid was used as a template to generate a DNA fragment for homologous recombination with BAC16 inside bacteria. (vi and vii) After confirmation of insertion at the correct site by colony PCR screening, the kanamycin cassette was deleted by recombination after induction of I-SceI in bacteria by l-arabinose. Correct insertion of the fluorescence tags and integrity of BAC DNA were confirmed by sequencing of PCR-amplified fragments and restriction digestions. (viii and ix) Additional viral proteins were similarly tagged with mCherry and mCardinal fluorescent protein. Primers and DNA fragment used are listed in Table 1.

Isolation of different stages of KSHV replicating cells from a dish can increase the resolution for a number of downstream analyses. To perform such studies in a convenient manner, we designed a reporter-KSHV that enables both visualization and isolation for cells of interest from the cell population by flow cytometer. We selected three viral genes with different expression kinetics for the following reasons: (i) ORF6 was selected because of its well-documented function in the establishment of replication compartments (RCs) in infected cells that should lead us to identify KSHV DNA replicating cells (39, 40); (ii) LANA was selected to visualize location and track copy number of latent KSHV episomes during cell proliferation (41); and (iii) ORF52 (tegument protein) was selected because of its higher expression during replication. Fusion of enhanced green fluorescent protein (EGFP) to the ORF52 N terminus also was shown to have little effect on the production of KSHV virions in culture media (42). Three fluorescence tags (mBFP2, mCherry, and mCardinal) were selected based on their relative brightness and separation of fluorescence emission peaks. GFP is also expressed from the EF1α promoter from the bacterial artificial chromosome 16 (BAC16) backbone (22). Since the cDNA sequences of mBFP2 (blue) and mCardinal (far-red) have 95% identity, we optimized codon usage of mCardinal to alter the DNA sequence without changing the amino acid sequence to prevent recombination between the two fluorescence tags (Table 1). Each fluorescence tag was inserted sequentially with a two-step red recombination approach and expressed as a fusion protein (Fig. 1). We reasoned that the expression of the fluorescence marker as a fusion protein from the respective endogenous promoter would report KSHV gene expression more closely to that of the natural KSHV replication cycles. Four individual colors and combinations of merged fluorescence produced more than 7 colors; therefore, we called the reporter-KSHV “Rainbow-KSHV.” We also prepared two types of mBFP2-ORF6 fusions (N-terminal fusion or C-terminal fusion) separately and compared efficacies of KSHV gene expression.

**TABLE 1:** Primers, plasmids, and gene block DNA sequences used for BAC16 recombination (5′→3′)

Primer, plasmid, or gene block	Sequence^*a*^
mTagBFP2 cassette template, Addgene 142374	
mTagBFP2 KpnI-S	AAAGGTACCATGGTGTCTAAGGGCGAAGAGCTGATTA
mTagBFP2 SacII-AS	AAACCGCGGTTAATTAAGCTTGTGCCCCAGTTTGCTAG
Template pEPkan-S, Addgene (plasmid 41017)	
mTagBFP2-Kan AccI-S	AAAGGTACCATTGTCTACTATGTGGACTACAGACTGGAAAGAATCAAGGAGGCCAACAACGAGACCTAGGGATAACAGGGTAATCGATTT
Kan AccI-AS	AAAGGTACCGTAGACGCCAGTGTTACAACCAATTAACC
mBFP2-ORF6N-FW	*AGGGGACTCTGCGCGCTTAAGCGCCAAGCCATTATACACACGGGTTTTTTGTTGTCTTGGCCAATCGTGTCTCC***ATGGTGTCTAAGGGCGAAGAGCTGATTA**
mBFP2-ORF6N-RV	*TAGAGGTACCCGCAGGGACCAGTGGGGGCCGCAGACCCAATATTTTCCTCGAGGGTTTGTGGTCCCTTTAGCGC***ATTAAGCTTGTGCCCCAGTTTGCTAG**
mBFP2-ORF6C-FW	*GCTTTTTCCGTCCCCGGGCGTCCCAAGCCTGACAGTGGGTAAAAAACGAAAAATCGCATCCCTGCTCTCTGACCTGGATTTG***GTGTCTAAGGGCGAAGAGCTGATTAAGG**
mBFP2-ORF6C-RV	*GGTCCATGGCTAGGGCTGACACATCGGCATAGACCGCCGCCAGTTCCTTTGCCATCGTTACGGGTACACAA***CTAATTAAGCTTGTGCCCCAGTTTGCTA**
mCardinal codon-optimized fragment (KpnI/SacII) cloned into pBS SK+ vector with Gibson assembly	CGACTCACTATAGGGCGAATTGGGTACCATGGTCAGCAAAGGCGAAGAGTTGATTAAGGAGAACATGCATATGAAACTGTATATGGAGGGGACTGTTAATAACCATCACTTCAAATGCACTACAGAAGGCGAAGGAAAGCCATATGAGGGCACTCAGACGCAACGGATTAAAGTTGTTGAGGGTGGACCTTTGCCCTTTGCCTTCGACATACTGGCAACATGCTTTATGTATGGCTCCAAGACATTTATTAACCATACACAAGGAATACCCGACTTTTTCAAACAAAGTTTCCCAGAAGGATTCACATGGGAGAGAGTGACGACTTATGAGGACGGGGGAGTCCTTACGGTAACCCAAGATACCTCATTGCAGGACGGGTGCCTCATTTACAACGTGAAACTTAGGGGCGTCAACTTCCCATCTAACGGGCCAGTGATGCAGAAAAAAACTCTTGGATGGGAAGCTACAACTGAGACGCTCTATCCTGCGGATGGCGGCCTGGAGGGCCGCTGTGATATGGCCCTTAAGCTGGTAGGCGGTGGACATCTCCATTGCAATCTCAAAACTACGTACAGAAGCAAAAAACCCGCCAAAAATCTGAAGATGCCAGGCGTCTACTTTGTCGATAGAAGGCTTGAGAGAATCAAGGAGGCGGATAACGAAACTTATGTCGAGCAACATGAAGTGGCGGTAGCCAGGTACTGCGACCTTCCCTCAAAGCTGGGTCATAAGTTGAATGGGATGGATGAACTGTACAAGTAACCGCGGTGGAGCTCCAGCTTTTGTTCC
Template pEPkan-S, Addgene (plasmid 41017)	
mCardinal Kan AccI-S	AAAGGTACCGTCTACTTTGTCGATAGAAGGCTTGAGAGAATCAAGGAGGCGGATAACGAAACTTATAGGGATAACAGGGTAATCGATTT
Kan AccI-AS	AAAGGTACCGTAGACGCCAGTGTTACAACCAATTAACC
mCardinal-ORF52N-FW	*AGCTGGGTATAAAGGAGGGATTTGGGCTTTTGTTGTGAGCATCACCAACACGTACATCTACGCGTACCTGAC***ATGGTCAGCAAAGGCGAAGAGT**
mCardinal-ORF52N-RV	*TCAATTGGCTTATCTTTGCGGTTAGGTCTTCCATCGTAAGGTCCTTTTTGGGTCTGCCCCTGGGCGCGGC***CTTGTACAGTTCATCCATCCCAT**
mCherry cassette template, Addgene 60954	
mCherry KpnI-S	AAAGGTACCATGGTGAGCAAGGGCGAGGAGGA
mCherry NotI-AS	AAAGCGGCCGCCTACTTGTACAGCTCGTCCATGCCGCCGGTGG
Template pEPkan-S, Addgene (plasmid 41017)	
mCherry-Kan PstI-S	AAACTGCAGGACGGCGAGTTCATCTACAAGGTGAAGCTGCGCGGCACCAACTTCCCCTCCGACTAGGGATAACAGGGTAATCGATTT
mCherry-Kan PstI-AS	AAACTGCAGCCAGTGTTACAACCAATTAACC
mCherry LANA N-Fw	*ACTGCCACCGCCTCCATAATTTTACTTTGGTTGTCAGACCAGATTTCCCGAGG***ATGGTGAGCAAGGGCGAGGAGG**
mCherry LANA N-Rv	*CCTCTCGTTAAGGGCGCGCCGGTGCTCCGTCCCGACCTCAGGCGCATTCCCGGTGGCGC*ACCACC**CTTGTACAGCTCGTCCATGCCGCCGGTGGAG**

a Restriction enzyme sites used for cloning are underlined. Italics indicate homology arms for recombination. Boldface indicates sequence annealed to cloned fluorescent coding sequence for amplification DNA fragment for recombination.

### Rainbow-KSHV gene expression.

**FIG 2:**
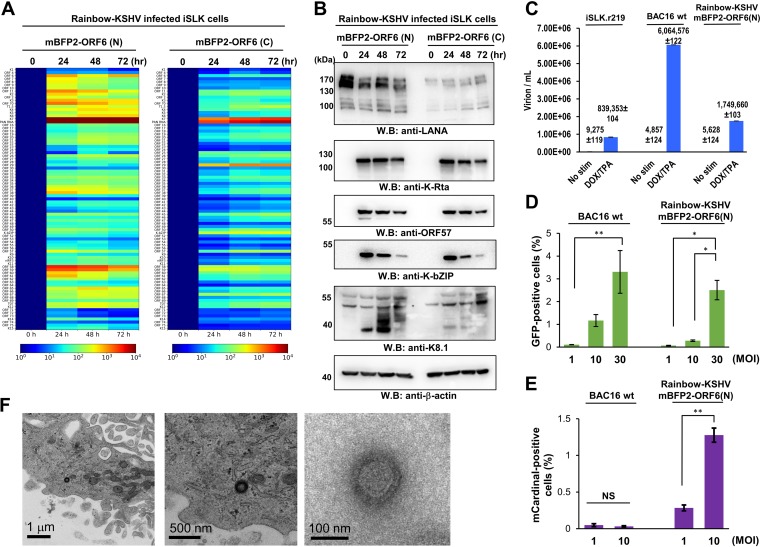
Viral gene expression and production of progeny virus. (A) Viral gene expression. Rainbow-KSHV-infected iSLK cells were stimulated with doxycycline and TPA. Total RNAs were purified at the indicated time points and subjected to KSHV PCR arrays. Gene expression is shown as a heatmap. 18S rRNA was used as an internal standard for normalization, and the 0-h time point was set as 1. (B) Viral protein expression. Total cell lysates were prepared at the indicated time points and subjected to immunoblotting. KSHV proteins and β-actin protein were probed with specific antibodies. WB, Western blotting. (C) Production of progeny virus. Capsidated viral DNA copy number was quantified by quantitative PCR 96 h poststimulation. (D) mCardinal fluorescence during initial infection. The indicated DNA copy numbers of virions were infected into iSLK cells, and the mCardinal-positive cell population was quantified with FACS at 4 h after infection. (E) *De novo* infection. The indicated DNA copy numbers of virions were infected into iSLK cells, and the GFP-positive cell population was quantified with FACS 48 h after infection. (F) Electron micrograph of viral particle. KSHV viral particles in reactivated cells (left and middle) and purified from tissue culture supernatant (right) were visualized by electron microscopy.

To establish Rainbow-KSHV-transfected iSLK cells, BAC16-Rainbow was directly transfected into iSLK cells and selected with a high concentration of hygromycin (1 mg/ml). Stably transfected cells were isolated and pooled to avoid clone variations. First, the expression of the entire viral gene was confirmed by KSHV PCR arrays after harvesting total RNAs at a different time point. All of the KSHV open reading frames (ORFs) and viral long noncoding RNAs (PAN RNA, T1.5, and T0.7) were induced in the presence of TPA and doxycycline. Although both the N-terminally and C-terminally tagged mBFP-ORF6 virus expressed every KSHV gene, N-terminal ORF6 showed lower overall threshold cycle (*C_T_*) values in the cells (Fig. 2A). Accordingly, we decided to use Rainbow-KSHV with N-terminal ORF6 iSLK cells for further analyses. Sodium butyrate and doxycycline also strongly induced reactivation, including expression of all KSHV ORF proteins (see Fig. S1 in the supplemental material). KSHV protein expression also was confirmed by immunoblotting (Fig. 2B). N-ORF6 was found to have higher LANA expression than the C-ORF6 construct, which suggests the presence of higher episome copy numbers.

### Rainbow-KSHV replication.

We next examined viral particle production in culture supernatant. The results showed a clear increase of KSHV virions in culture media after stimulation. Rainbow-KSHV produces amounts of virions in supernatant comparable to those of rKSHV2.19 and about one-fourth of that produced by BAC16 wild-type virus (Fig. 2C). *De novo* infection of the iSLK cells released mCardinal-ORF52, a tegument protein, and therefore increased the number of mCardinal-positive cells. Numbers of GFP-positive cells were also increased in a dose-dependent manner, and the infection ratio was comparable with that of the BAC16 wild-type virus (Fig. 2D and E). Under electron microscopy, we observed densely stained virus-like particles in both reactivated cells and purified supernatant (Fig. 2F). Together, these results confirmed that fluorescence protein tags did not significantly interfere with viral DNA replication or virion production.

### Dynamic expression of fluorescence-tagged protein.

**FIG 3:**
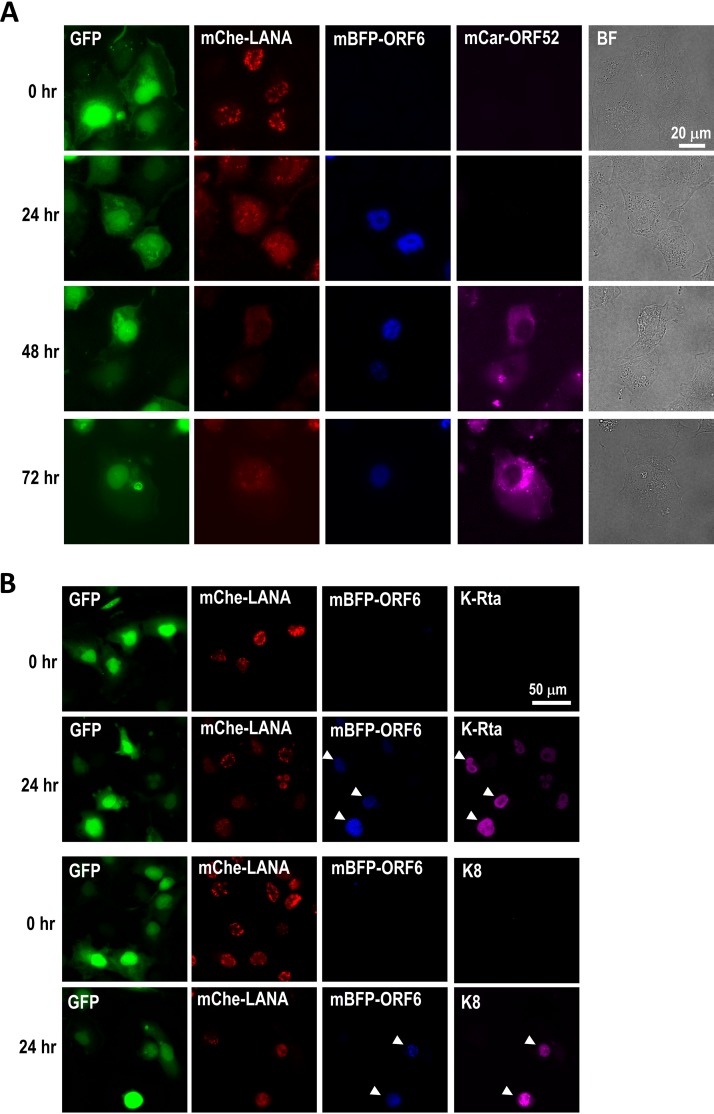
(A) Expression and localization of mCherry-LANA, mBFP-ORF6, and mCardinal-ORF52. iSLK cells latently infected with Rainbow-KSHV were stimulated with doxycycline and TPA for 0, 24, 48, and 72 h. The cells were fixed with 4% paraformaldehyde. Fluorescence protein-tagged viral proteins were visualized by fluorescence microscopy. BF, bright field. (B) K-Rta and K8 protein expression in Rainbow-KSHV-infected iSLK cells. Cells were fixed at 24 h after reactivation, and K8 and K-Rta were stained with specific antibody. Because most cells do not express mCardinal-ORF52 at the 24-h time point, we used Alexa647-conjugated secondary antibody for visualization of K-Rta and K8 proteins.

**FIG 4:**
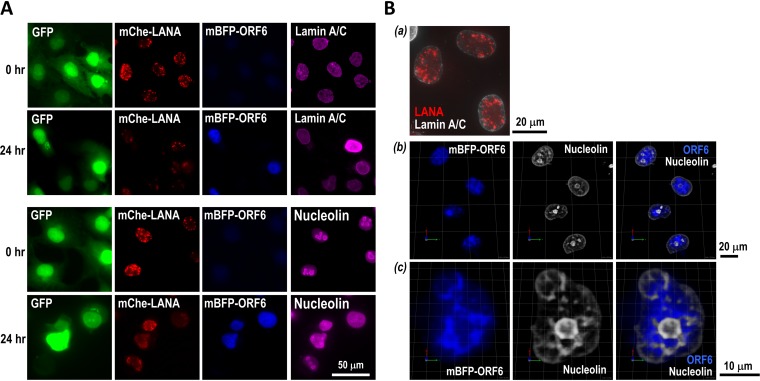
Spatial association of KSHV episome with nucleoli and nuclear envelope. (A) Immunofluorescence staining. Nuclear structure was visualized by immunofluorescence staining of lamin A/C and nucleolin in Rainbow-KSHV-infected iSLK cells at the indicated time points after stimulation. (B) 3D reconstruction of images. The Z-stack images were reconstructed as 3D images with Volocity software. After 24 h of stimulation, mBFP-ORF6 expression (blue) appears to fill the interchromosomal space surrounding the host cell nucleoli, marked by antinucleolin staining (greyscale).

We next reactivated the cells for different time points, and we fixed and observed fluorescence signals *in situ*. The mCherry-LANA fusion had unique nuclear localization patterns, known as LANA dots, before reactivation (Fig. 3A). The red fluorescent protein (RFP)-LANA-encoding recombinant KSHV was recently reported by Lieberman’s group; our Rainbow-KSHV showed similar LANA expression patterns (41). The majority of the LANA dots were peripherally distributed, just inside the nuclear membrane or surrounding the nucleoli (Fig. 4), and were relatively immobile over the course of live cell imaging. However, those LANA dots started to diffuse after triggering reactivation (Fig. 3A). At 24 h after stimulation, mBFP-ORF6 became visible and some mBFP-ORF6 showed unique puncta, while others showed very bright spots. The bright dot-like structures were not a result of the accumulation of multiple smaller puncta; the time lapse showed that the mBFP-ORF6 dots appeared as dots from the beginning (Fig. S2). The LANA dots and mBFP-ORF6 did not colocalize with each other. At 48 h postinduction, mCardinal-ORF52 became visible and was expressed primarily in the cytoplasm. Strong signals of mCardinal-ORF52, however, did not correlate with the presence of brighter mBFP2 signals (Fig. 3A). The mCardinal-ORF52 signal intensities gradually increased, while both LANA dots and mBFP2-ORF6 signals were usually absent from those ORF52-positive cells (Fig. 3A, row for 72 h). Where specific antibodies were available to us, immunoblotting or immunofluorescence staining confirmed the identity of the fusion proteins. mCardinal-ORF52 protein was detected as an approximately 40-kDa protein in both ORF52 and RFP antibodies (Fig. S3A). The distribution of mCherry fluorescence also was directly correlated with LANA antibody staining (Fig. S3B). These results indicate that tagged fusion proteins were not cleaved and indeed were intact. K-Rta expression and expression of another early protein (K8) were used to mark reactivating cells by immunofluorescence staining at 24 h before the majority of mCardinal-ORF52 expression. Because of exogenous K-Rta expression from the tet-inducible cassette, K-Rta was present in every cell, while a few cells showed stronger signals. This is presumably due to additional K-Rta expression from Rainbow-KSHV genomes (Fig. 3B). K8 expression was present only in mBFP-ORF6-positive cells, ensuring mBFP2-positive cells are the reactivating cells (Fig. 3B).

### Subcellular localization of KSHV episomes and KSHV replication compartments in the host cell nucleus.

To better understand how KSHV episomes reorganize during reactivation to form viral replication compartments inside the host cell nucleus, we visualized nuclear membrane and nucleoli by labeling lamin A/C and nucleolin, respectively. Fluorescence-labeled KSHV episomes were frequently observed in proximity to these structures but never directly on the nuclear membrane or in nucleoli (Fig. 4A and B). During reactivation, mBFP2-ORF6, which is associated with KSHV replication compartments, appeared to accumulate around nucleoli and partially colocalize with nucleolin (Fig. 4B). The nucleolus structure also was disrupted in mBFP2-positive cells, and nucleolin appeared to leak into the nucleoplasm (Fig. 4A, bottom, 0 h versus 24 h). High-magnification three-dimensional (3D) images showed the invasion of nucleoplasmic space by the mBFP2-ORF6 protein (Fig. 4B).

### Studying reactivation kinetics in cell populations at single-cell resolution.

**FIG 5:**
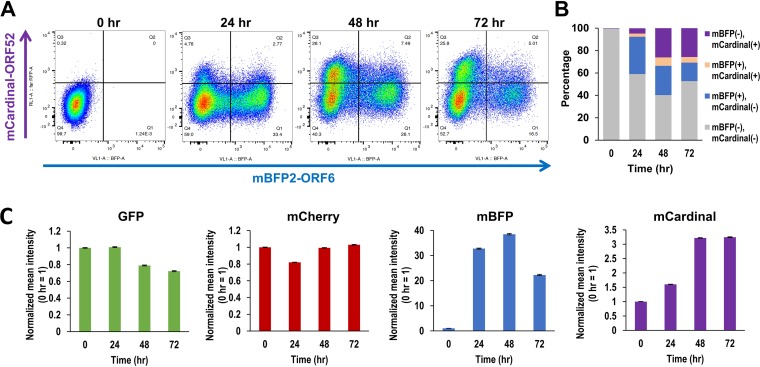
Flow cytometry analysis of Rainbow-KSHV-infected cells. (A) Profile of mBFP-ORF6 and mCardinal-ORF52 expression after stimulation. iSLK cells infected with Rainbow-KSHV were stimulated with doxycycline and TPA. The cells were fixed with 2% paraformaldehyde at the indicated time point, and expression of GFP, mCherry-LANA, mBFP-ORF6, and mCardinal-ORF52 was analyzed by flow cytometry. Gating strategies are summarized in the supplemental material (Fig. S4). (B) Combination of fluorescence. Percentages of cell populations with fluorescence-positive and -negative cells are shown in the bar chart. (C) Mean intensities of each fluorescence protein. Changes of mean intensities were calculated. Fluorescence intensity at the 0-h time point was set to 1.

We next monitored KSHV reactivation by flow cytometry. iSLK-Rainbow was induced with doxycycline and TPA for 8 h, and cells were harvested at 24-h intervals for 3 days. The proportion of fluorescent-positive cells, fluorescence intensities, and their color combinations in reactivating cells were monitored. As shown in Fig. 5, intensity and number of mBFP-positive cells were strongly increased at 24 h, further increased until 48 h, and then declined by the 72-h time point. The mCardinal signals gradually accumulated at 24 h and reached the highest number and intensity at 48 h. The signal then stayed at similar levels until the end of the time period. Considering that a fraction of cells died off and were eliminated from the analyses by a gating strategy (Fig. S4), the result suggested that mCardinal-ORF52, but not mBFP-ORF6, continued to express and maintained a proportion of positive cells between 48 and 72 h. The LIVE/DEAD staining confirmed that approximately 30% of cells died in the first 24 h of reactivation, presumably due to the toxicity of the stimuli (Fig. S5). A smaller fraction of the cell population was positive for both mBFP2 and mCardinal (5.0%) than of cells expressing either mBFP (16.5%) or mCardinal alone (25.8%) at 72 h (Fig. 5B), suggesting rapid elimination of mBFP2-ORF6 protein in mCardinal-expressing cells. Time-lapse live imaging showed that approximately 10% of overall mBFP-ORF6-positive cells expressed mCardinal-ORF52 within a 12-h time frame, indicating there is a hurdle for viral late protein expression, at least for the amount of mCardinal visible under fluorescence microscopy. At the same time, small numbers of cells rapidly expressed mCardinal in fewer than 24 h (Fig. 5A and B). These results suggested a wide range of kinetics of KSHV gene expression and DNA replication at the individual cell level. Even though most LANA dots appeared to diffuse after 24 h of reactivation (Fig. 3), the overall mCherry signal intensity in each cell was relatively unchanged (Fig. 5C), suggesting LANA was dissociated from the TR sequence and diffused to the nucleoplasm without degradation. Similar kinetics and phenotypes were also seen in 293FT cells, albeit at much lower rates of reactivation (Fig. S6).

### Inhibition of mBFP2-ORF6 gene expression in the presence of mCardinal-ORF52.

**FIG 6:**
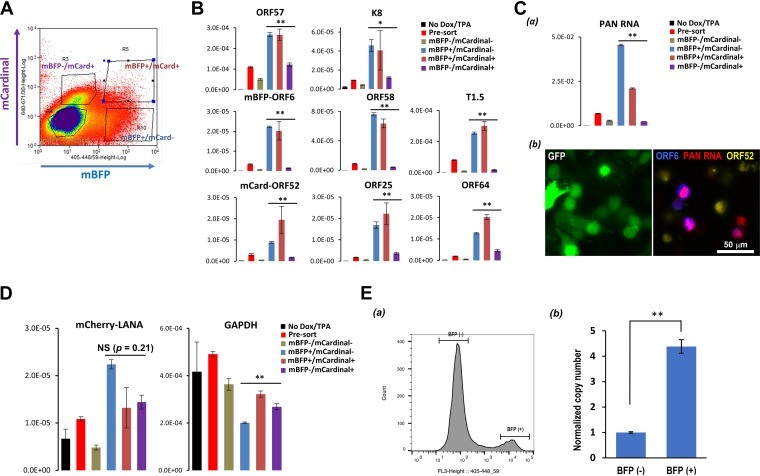
Fractionation of reactivated cells from nonresponding cells. (A) Reactivated cell fractions by color codes. Reactivated cells were fractionated with FACS into four populations: (i) mBFP^−^ mCardinal^−^, (ii) mBFP^+^ mCardinal^−^, (iii) mBFP^+^ mCardinal^+^, and (iv) mBFP^−^ mCardinal^+^. (B) Heterogenic viral gene expression at a given time point. Total RNA was extracted from fractionated cells, and expression of latent, immediate-early, early, and late lytic genes was assessed by real-time qPCR and shown as bar graphs. 18S rRNA was an internal standard for normalization. Black, red, gray, blue, orange, and purple columns represent 0-h presort, 48-h presort, 48-h mBFP^−^ mCardinal^−^, 48-h mBFP^+^ mCardinal^−^, 48-h mBFP^+^ mCardinal^+^, and 48-h mBFP^−^ mCardinal^+^, respectively. ANOVA followed by Tukey’s test was performed for 48-h mBFP^+^ mCardinal^−^, 48-h mBFP^+^ mCardinal^+^, and 48-h mBFP^−^ mCardinal^+^. *, *P* < 0.05. **, *P* < 0.01. (C) Expression of PAN RNA. PAN RNA expression was examined with real-time qPCR (a) and RNA-FISH (b). Red, PAN RNA; blue, mBFP2-ORF6; yellow, mCardinal-ORF52. ANOVA followed by Tukey’s test was performed for 48-h mBFP^+^ mCardinal^−^, 48-h mBFP^+^ mCardinal^+^, and 48-h mBFP^−^ mCardinal^+^. **, *P* < 0.01. NS, not significant. (D) Expression of mCherry and GAPDH at a given time point. (E) Heterogenic viral DNA replication. iSLK cells latently infected with Rainbow-KSHV were stimulated with doxycycline and TPA for 24 h. Cells were fractionated by FACS into two populations: BFP^−^ (i) and BFP^+^ (ii). DNA was purified from each cell fraction, and viral DNA copy number was determined by real-time qPCR. **, *P* < 0.01.

The schematic presented in Fig. 6A shows our strategy of cell fractionation. KSHV PCR arrays were employed to analyze KSHV gene expression for each of the cell pools, and selected viral genes with different kinetics were shown (Fig. 6B). As expected, isolating reactivating cells from nonresponders increased dynamic ranges of viral gene expression approximately 5- to 10-fold. Isolation of cells most strongly lowered *C_T_* values of late genes; this likely reflects the smaller proportion of late gene-expressing cells in the cell population. Interestingly, a cell fraction (mCard^+^/mBFP^−^) showed very little late gene expression despite the presence of mCardinal protein, indicating late gene expression occurs very transiently and happens spontaneously with viral DNA replication. The largest amount of late gene expression was indeed seen in the double-positive cell fraction (mCard^+^/mBFP2^+^). With fractionation, we also observed clear downregulation of most of the viral genes in the mCardinal^+^/mBFP^−^ cell fraction. The inclusion of proteasome or lysosomal inhibitors did not recover mBFP2 expression in the mCardinal-positive fraction, suggesting that downregulation of mBFP2 was primarily at transcriptional levels (Fig. S7). Downregulation of early genes in mCardinal-positive cells was further confirmed by RNA-fluorescent *in situ* hybridization (FISH) with PAN RNA probes. Strong PAN RNA signals (red) were observed mostly in the mBFP-ORF6-positive cells (blue). Consistent with quantitative PCR (qPCR) results, mCardinal-positive cells (yellow) showed much lower intensities of PAN RNA signals (Fig. 6C and Fig. S8). Of note, the RNA-FISH signal of PAN RNA was extremely strong, so the exposure time for the red channel was set to 1/100 s. With this setting, we avoided having overlapping signals from mCherry-LANA, which requires a minimum of 0.5 s of exposure with the same setting. Although differences of cellular glyceraldehyde-3-phosphate dehydrogenase (GAPDH) expression among three groups (blue, brown, and purple; mBFP- and/or mCardinal-positive fractions) were statistically significant (*P* < 0.01), those fold changes were much smaller (less than 1.5 times) than those of viral lytic genes in the mCardinal-positive fraction, especially in the mCardinal-only group (Fig. 6B and C, purple bars). Similar regulation was not seen in LANA expression. The result suggests the presence of a specific molecular mechanism to regulate inducible lytic gene expression. We also confirmed higher numbers of intracellular viral DNA copies in the mBFP-positive cell fraction at 24 h (Fig. 6E). These results together demonstrated increased resolution of analyses.

## DISCUSSION

Genetic and molecular heterogeneity in cells of the same tissue type and lineage has become increasingly evident through advanced single-cell omics (43, 44). Whether it originates from spontaneous changes in gene expression or stochastic responses to environmental perturbations, this inherent heterogeneity limits the potential efficacy of targeted therapeutics. Similarly, in our research, we believe that molecular heterogeneity accounts for the wide range of responses to KSHV reactivation stimulation. It has been known that lytic proteins, particularly those of late genes, are not uniformly expressed, even after strong stimulation by K-Rta and TPA treatment. We would like to understand the differences that lead some cells to respond and others to be nonresponders or abortive responders under the same experimental conditions. However, it is practically impossible to use conventional methods to identify molecular mechanisms when samples inevitably contain mixtures of cells exhibiting these different behaviors. Therefore, we developed Rainbow-KSHV as a tool for visualization and spot determination of the KSHV reactivation stage in live host cells.

Recent single-cell transcriptome analysis techniques have indeed revealed that viral infection and replication is highly heterogeneous, with only a small fraction of infected cells producing the bulk of progeny virus. Russell et al. recently reported that there is extremely large cell-to-cell variation in the production of transcription in influenza virus-infected cells (45). Similar transcriptional heterogeneity was also reported in HSV-1-infected cells (46). In KSHV infection, several reports demonstrated abortive infection, in which only selected viral genes are expressed temporally and do not produce viral particles. Infection of nonpermissive primary cells or deletion of the K8 gene from the KSHV genome also led to abortive infection (47, 48). In addition, one of the key cellular factors required for the completion of KSHV replication in B cells has been identified. CBF1/CSL, which plays a role in stronger and/or continuous induction of lytic gene expression, is essential for completion of KSHV replication, and deletion of the host gene from B cells led to abortive infection (49). These studies clearly suggest completion of KSHV replication hinges on fine balances of the degree of activation of viral genomes (e.g., viral DNA episome numbers, amount of K-Rta protein expression via strength, duration of K-Rta promoter activation, and presence of cellular cofactors/activator) (24, 50–52) and condensation of KSHV genomes by the viral and host factors (53–56). Higher resolutions of analyses will be increasingly important to identify host/viral factors associated with such outcomes.

It is important to note that Brulois et al. previously generated a similar reporter construct, named RGB-BAC16 (red-green-blue-BAC16). In this reporter virus, expression of mRFP1 (red), EGFP (green), and tagBFP (blue) was placed under the control of constitutively active EF1α, a PAN RNA promoter, and a K8.1 promoter, respectively (57). Differences between our reporter construct and the RGB-BAC16 are that (i) the expression of fusion proteins from the endogenous corresponding promoters is expected to report viral gene expression more closely resembling that of the natural settings of viral replication, (ii) latent viral copy number in each infected cell can be measured by counting LANA dots in the nucleus, (iii) the establishment of a viral replication compartment may be visualized in live cells with mBFP-ORF6 fluorescence, which is directly associated with KSHV DNA replication, and (iv) fluorescence intensity directly correlates with the amount of corresponding viral protein in the cells. Nonetheless, the RGB-BAC16 has been used widely in many laboratories with great success (30, 58–60), and we are hopeful that Rainbow-KSHV will become an important complement.

During the course of our studies, we realized that iSLK cells produced the largest amount of DNase I-resistant viral DNA; however, we also noticed that a higher viral DNA copy number in culture media did not always correspond to a higher infection rate after *de novo* infection. Based on our detection and measurement of mCardinal fluorescence, we think only a small fraction of cells produce viral particles, and of these, an even smaller subpopulation are generating infectious particles in the culture supernatant. It is interesting to determine specific cell types and tissue culture conditions that favor producing more infectious viral particles in the supernatant; single-cell analyses with proteomics approaches may help to answer the question.

The unique staining pattern of mBFP2-ORF6, particularly in relation to that of nucleolin, a protein maker for the nucleolus, is interesting and possibly significant. The nucleolus has the most active cellular transcription site in the nucleus but also has an attractive compartment for nuclear heterochromatic regions, such as pericentric repeats and regions enriched in repressed genes with low gene density (61, 62). Thus, the nuclear space is the region for the coexistence of euchromatic and heterochromatic rRNA genes. Interestingly, cytomegalovirus UL84 and UL44 proteins have been shown to interact with nucleolin and play a role in the formation of viral replication compartments near the nucleoli (63, 64). For EBV, EBNA1 interacts with nucleolin, while the RNA binding domain of nucleolin plays a critical role in EBV episome maintenance (65). We recently found that KSHV episomes preferentially tether near the centromere of the host chromosome and other nucleolus-associated chromosomal domains (66 and M. Campbell and Y. Izumiya, unpublished observations). We postulate that the unique nuclear environment is suitable for establishment/maintenance as well as reactivation of KSHV after stimulation.

Another notable finding is that small cell populations support KSHV replication very well. About 4% of iSLK cells have shown mCardinal signals at the 24-h time point. In light of HSV-1 replication kinetics with a high multiplicity of infection (MOI), it is not surprising that KSHV can replicate in a short period of time. The result is also consistent with quantitative reverse transcription-PCR (qRT-PCR) array studies that have shown lowered *C_T_* values of late genes 24 h postactivation (Fig. 2A). What makes the cellular environment ideal for viral replication requires further investigation. In HSV-1 studies with ICP0 mutant virus, it was demonstrated that there is a threshold at which the mutant virus can replicate normally (67). Those studies indicated that increased MOI overcame defects of the mutations. A cell with robust induction of KSHV gene expression may enter KSHV replication, while other cells might need extra time to accumulate viral transcriptional factors to pass a certain threshold. The activity of specific transcriptional factors in the cell or epigenetic gene modifications, including a genomic structure, are likely the key factors for efficient RNA polymerase II recruitment. In addition, a high latent episome copy number likely would increase the chance of producing a higher yield. By counting LANA dots in a 3D nucleus, we also noticed that the KSHV episome number changed with every cell division, which also may be associated with the heterogenic responses to the stimuli. Combinations of mBFP2-ORF6 also can offer information for when, in which cells, and where in the nucleus KSHV starts to replicate. We think this Rainbow-KSHV is an especially powerful tool for analyzing the association of KSHV replication with cellular differentiation in a 3D organotypic culture system or organoid tissue culture models.

By fractionating infected cells by replication stages, we could demonstrate clear temporal viral gene regulation. KSHV lytic gene expression was strongly diminished in the mCardinal^+^/mBFP2^−^ cell fraction. Interestingly, such downregulation was more clearly seen in lytic genes (mostly early and late genes). More late gene transcripts in the mBFP2-positive fractions than mBFP2-negative cell population also suggests transient late gene expression in mBFP2-positive (DNA-replicating) cells. Late gene expression in the double-positive (mBFP2^+^/mCardinal^+^) cell fraction showed a *C_T_* value comparable to that of immediate-early and early genes. Accordingly, seemingly lower level of expression of late genes in previous studies likely was due to lower cell numbers within the cell population that successfully entered late viral replication stages, as well as the shorter window of their expression kinetics. Finally, we are hopeful that our Rainbow-KSHV contributes to rapidly developing single-cell approaches and further advances our understanding of KSHV replication cycles. With single-cell genomics and proteomics approaches with Rainbow-KSHV, we may be able to probe key activators and/or more of the restriction factors for KSHV replication in the near future.

## MATERIALS AND METHODS

### Chemicals.

Dulbecco’s modified minimal essential medium (DMEM), fetal bovine serum (FBS), phosphate-buffered saline (PBS), Trypsin-EDTA solution, 100× penicillin–streptomycin–l-glutamine solution, anti-RFP antibody, Alexa 647-conjugated secondary antibody, SlowFade Gold antifade reagent, and a LIVE/DEAD fixable yellow dead cell stain kit were purchased from Thermo Fisher (Waltham, MA USA). Puromycin solution and G418 solution were obtained from InvivoGen (San Diego, CA, USA). Hygromycin B solution was purchased from Enzo Life Science (Farmingdale, NY, USA). Anti-ORF57, anti-K8, and anti-K8.1 antibodies were purchased from Santa Cruz Biotechnology, Inc. (Santa Cruz, CA, USA). Anti-LANA and anti-lamin A/C antibodies were purchased from Millipore-Sigma (St. Louis, MO, USA). Anti-nucleolin antibody was purchased from Abcam (Cambridge, UK). Anti-K-Rta antibody was described previously (68). Monoclonal anti-ORF52 antibody was a generous gift from Fanxiu Zhu (Florida State University). All other chemicals were purchased from Millipore-Sigma (St. Louis, MO, USA) unless otherwise stated.

### Cell culture.

iSLK.219 cells were maintained in DMEM supplemented with 10% FBS, 10 μg/ml puromycin, 400 μg/ml hygromycin B, and 250 μg/ml G418. iSLK cells were obtained from Don Ganem (Novartis Institutes for Biomedical Research). 293 (ATCC, Manassas, VA, USA) and 293T (ATCC, Manassas, VA, USA) cells were grown in DMEM containing 10% FBS.

### Construction of Rainbow-KSHV BAC16.

Recombinant KSHV was prepared by following a protocol for *en passant* mutagenesis with a two-step markerless red recombination technique (69). Briefly, fluorescence protein-coding sequences first were cloned into a pBS SK vector (Thermo Fisher, Waltham, MA, USA) after amplifying cDNA from the respective plasmid as templates, obtained from Addgene (Table 1). The pEPkan-S plasmid was also used as a source of the kanamycin cassette, which includes the I-SecI restriction enzyme site at the 5′ end of the kanamycin coding region (69). The kanamycin cassette was amplified with primer pairs listed in Table 1. An amplified kanamycin cassette then was cloned into the fluorescence protein-coding region at a unique restriction enzyme site (e.g., AccI for mBFP2 and mCardinal, PstI for mCherry). The resulting plasmid was used as a template for another round of PCR to prepare a transfer DNA fragment for markerless recombination with BAC16 (22). Recombinant BAC clones with insertion and also deletion of the kanamycin cassette in the BAC16 genome were confirmed by colony PCR with appropriate primer pairs. Recombination junctions and adjacent genomic regions were amplified by PCR, and the resulting PCR products were directly sequenced with the same primers to confirm in-frame insertion into the BAC DNA. The resulting recombinant BAC was also confirmed by restriction enzyme digestions (HindIII and BglII) if there were any large DNA deletions. Two independent BAC clones were generated for each fluorescence-tagged recombinant virus as biological replicates.

### Western blotting.

Cells were lysed in immunoprecipitation lysis buffer (25 mM Tris-HCl, pH 7.4, 150 mM NaCl, 1% NP-40, 1 mM EDTA, 5% glycerol) containing protease inhibitors (Roche, Basel, Switzerland). Total cell lysates (25 μg) were boiled in SDS-PAGE loading buffer, subjected to SDS-PAGE, and subsequently transferred to a polyvinylidene fluoride membrane (Millipore-Sigma, St. Louis, MO, USA) using a semidry transfer apparatus (Bio-Rad, Hercules, CA, USA). The final dilution of the primary antibody was 1:5,000 for anti-K-Rta rabbit serum, 1 μg/ml anti-K8α (Santa Cruz, Santa Cruz, CA, USA), 1 μg/ml anti-ORF57 mouse monoclonal antibody (Santa Cruz, Santa Cruz, CA, USA) and anti-LANA rat monoclonal (Millipore-Sigma, St. Louis, MO, USA), 1 μg/ml anti-K8.1 mouse monoclonal (Santa Cruz, Santa Cruz, CA, USA), 1:1,000 anti-RFP monoclonal antibody (Thermo Fisher), 1:500 anti-ORF52 monoclonal antibody, and 1:5,000 anti-β-actin mouse monoclonal (Millipore-Sigma, St. Louis, MO, USA). Washing membranes and secondary antibody incubations were performed as described previously (50).

### Immunofluorescence microscopy and 3D image analysis.

iSLK cells latently infected with Rainbow-KSHV were seeded onto glass coverslips 20 to 24 h before stimulation. The cells then were stimulated with 1 μg/ml doxycycline and 20 ng/ml 12-*O*-tetradecanoylphorbol 13-acetate (TPA) for 0, 24, 48, and 72 h. The stimulated cells were fixed with 4% paraformaldehyde, permeabilized with 0.2% Triton X-100 in PBS, and incubated with 2% bovine serum albumin (BSA) in PBS to block nonspecific antibody binding. The cells then were treated with the primary antibody followed by fluorescent dye-conjugated secondary antibody. Cell imaging was performed on a Keyence BZ-X700 inverted microscope (Keyence, Osaka, Japan). Fluorophores and filters used in this study are summarized in Table 2. All filters were purchased from Chroma Technology Co. (Bellows Falls, VT, USA). For 3D visualization and analysis, Z stack image data were converted to greyscale in TIF format and imported to Volocity (Quorum Technologies, Inc., ON, Canada). Fluorophores and filters used in this study are summarized in Table 2.

**TABLE 2:** Fluorophores and filters used in this study

Fluorophore	Excitation (nm)	Emission (nm)	Filter (nm; excitation, emission)
EGFP	488	507	470/40, 525/50
mCherry	587	610	545/25, 605/70
mTagBFP2	399	454	360/40, 460/50
mCardinal	604	659	640/30, 700/75
Alexa 647	650	670	640/30, 700/75
Quasar 570	548	570	545/25, 605/70

### RNA-FISH.

iSLK cells infected with Rainbow-KSHV were seeded onto glass coverslips and reactivated with 1 μg/ml doxycycline and 20 ng/ml TPA for 48 h. Cells were fixed with 4% formaldehyde–PBS. Coverslips were incubated with 100 mM glycine-PBS for 10 min and washed with PBS three times in a 6-well plate. Cells were treated with 0.2% Triton-X and 0.05% SDS for 10 min and subsequently washed with FISH wash buffer (10% formamide [Millipore-Sigma, St. Louis, MO, USA], 2× SSC [1× SSC is 0.15 M NaCl and 0.015 M sodium citrate]) three times. PAN RNA FISH probes were hybridized on glass slides in hybridization buffer (10% dextran sulfate, 100 μg/ml yeast tRNA [Millipore-Sigma, St. Louis, MO, USA], 10% formamide, 125 nM FISH probe) overnight at 37°C. Cover slips were washed with FISH wash buffer and mounted with SlowFade Gold antifade reagent. Probe sequences are listed in Table 3 and were obtained from LGC Biosearch Technologies (Teddington, UK). More detailed step-by-step methods were published previously (70).

**TABLE 3:** RNA-FISH probe sequence for PAN RNA^*a*^

Probe	Sequence
PAN RNA probe_1	AAGAAGGCAAGCAGCGAGCA
PAN RNA probe_2	CGGCACCAATGAAAACCAGA
PAN RNA probe_3	GACGCAATCAACCCACAATC
PAN RNA probe_4	CAGGATGGGTATATTGCCAA
PAN RNA probe_5	GACGGAGAACCTAGCCGAAA
PAN RNA probe_6	CAGGCCAATGTGGGAAAAGT
PAN RNA probe_7	GCGGTGTTTTTTGTACTACA
PAN RNA probe_8	TTTTGTTCTGCGGGCTTATG
PAN RNA probe_9	AGGTTTTTGGGCAAATCGCA
PAN RNA probe_10	TGACTGTATAGTTGCCATGG
PAN RNA probe_11	CAATGCAATAACCCGCAAGG
PAN RNA probe_12	CAACTGGCCTGGAGATTGAA
PAN RNA probe_13	CGCATATCATAAAAGGGGGC
PAN RNA probe_14	TCCACATTCAGACACGTTAA
PAN RNA probe_15	CTGCTTTCCTTTCACATTAT
PAN RNA probe_16	ACTGTTCTGATACACCAGTG
PAN RNA probe_17	ATGAGCAGATAGGTAGTGCA
PAN RNA probe_18	TCAGACAAACACAGAACCGA
PAN RNA probe_19	ATCCGCTTTCTAGAATTACC
PAN RNA probe_20	CAAAGTGGCCCGATTTACAC
PAN RNA probe_21	ACACATCCAGATTGTCACAT
PAN RNA probe_22	AAATGCTTCACAACGCACCA
PAN RNA probe_23	ATTACAGCACTAGCCTGATA
PAN RNA probe_24	GTCATTCAAATCGACTTGCT
PAN RNA probe_25	GAAACTTCTGACAAATGCCA
PAN RNA probe_26	CACACCACTTTAGTCCAATG
PAN RNA probe_27	AACATTGAAAGAGCGCTCCC
PAN RNA probe_28	ACATCGTTAGTCAACCTAGC
PAN RNA probe_29	CACAACGCTTTCACCTACAA
PAN RNA probe_30	CAGCCAAAACACCGTTATCA
PAN RNA probe_31	GTCCGGTGCGAACAAGGAAA
PAN RNA probe_32	TGAGCTCTAGGCACGTTAAA
PAN RNA probe_33	AGTTGCATTACGTTATGGTA

a All oligonucleotides were labeled with Quasar 570 and pooled for RNA-FISH.

### Quantification of viral copy numbers.

Two hundred microliters of cell culture supernatant was treated with 12 μg/ml DNase I for 15 min at room temperature to degrade DNAs that are not correctly encapsidated. This reaction was stopped by the addition of EDTA to 5 mM, followed by heating at 70°C for 15 min. Viral genomic DNA was purified using a QIAamp DNA minikit according to the manufacturer’s protocol and eluted in 100 μl of AE buffer. Two microliters of eluate was used for real-time qPCR to determine viral copy number, as described previously (50).

### Preparation of purified KSHV.

iSLK cells latently infected with Rainbow-KSHV were seeded in eight to ten 15-cm dishes, stimulated with 1 μg/ml doxycycline and 20 ng/ml TPA for 24 h, and further incubated with culture media without stimuli for 72 h. The culture supernatant was centrifuged using a Beckman SW28 rotor (25,000 rpm for 2 h) with a 25% sucrose cushion. Virus pellet was dissolved in DMEM and further purified by discontinuous sucrose gradient (25 to 60%) centrifugation using a Beckman SW40Ti rotor (21,000 rpm, for 16 h). The virus pellet was dissolved in DMEM (for infection) or in water (for electron microscopy). Viral copy number was determined by real-time qPCR as described elsewhere.

### Real-time RT-PCR.

Total RNA was isolated using a quick RNA miniprep kit (Zymo Research, Irvine, CA, USA). First-strand cDNA was synthesized using a high-capacity cDNA reverse transcription kit (Thermo Fisher, Waltham, MA USA). Gene expression was analyzed by real-time qPCR using specific primers for KSHV ORFs designed by Fakhari and Dittmer (71). We used 18S rRNA as an internal standard to normalize viral gene expression.

### FACS analysis.

iSLK cells latently infected with Rainbow-KSHV were seeded in 6-cm dishes and stimulated with 1 μg/ml doxycycline and 20 ng/ml TPA for 0, 24, 48, and 72 h. Stimulated cells were fixed with 2% paraformaldehyde and suspended in 1% BSA in PBS. Samples were acquired on an Attune flow cytometer (Thermo Fisher, Waltham, MA USA) and analyzed with FlowJo software (BD Bioscience, San Jose, CA, USA). For live/dead fluorescence-activated cell sorting (FACS) analysis, stimulated cells were stained with LIVE/DEAD fixable yellow dead cell stain kit according to the manufacturer’s protocol. The cells then were fixed and analyzed as described above.

Alternatively, 293FT cells infected with Rainbow-KSHV were seeded in 6-cm dishes and stimulated with 1.5 mM sodium butyrate and 20 ng/ml TPA for 0, 24, 48, and 72 h. Stimulated cells were fixed and analyzed with a flow cytometer as described above.

### Cell fractionation using FACS.

iSLK cells harboring Rainbow-KSHV were seeded in 10-cm dishes and stimulated with 1 μg/ml doxycycline and 20 ng/ml TPA for 0, 24, and 48 h. Cells were trypsinized and suspended in 1% BSA in PBS. Cell fractionation was performed using a MoFlo Astrios EQ cell sorter (Beckman Coulter, Indianapolis, IN, USA) or InFlux cell sorter (Becton, Dickinson, Franklin Lakes, NJ) for subpopulations based on fluorescence codes. DNA or total RNA was extracted using a QIAamp DNA minikit (Qiagen, Venlo, Netherlands) or quick-RNA miniprep kit (Zymo Research, Irvine, CA, USA), respectively, immediately after cell fractionation.

### Electron microscopy.

Isolated virions were visualized by negative staining on TEM. Briefly, small droplets (∼10 μl) of purified KSHV in water were placed on 400-mesh copper grids with Formvar/carbon support film (Ted Pella, Inc., Redding, CA, USA) and left in a dust-free environment for 10 min. After wicking off the excess moisture with filter paper, 10 μl of 2% uranyl acetate in distilled H_2_O was added to the grid and immediately removed (again by wicking with filter paper). Grids were allowed to air dry completely before viewing by transmission electron microscopy (FEI Talos L120C equipped with an integrated Ceta CMOS camera; Thermo Fisher, Waltham, MA USA) at 100 kV.

iSLK cells latently infected with Rainbow-KSHV were stimulated with 1 μg/ml doxycycline and 20 ng/ml TPA for 72 h. Cells were collected and fixed in modified Karnovsky solution containing 2.5% glutaraldehyde and 2% paraformaldehyde in 0.1 M sodium phosphate buffer. Cells were subsequently rinsed twice in 0.1 M NaH_2_PO_4_ for a total of 30 min and resuspended in 0.1 M NaH_2_PO_4_ containing 1% osmium tetroxide and 1.5% potassium ferrocyanide for 45 min. Cells again were rinsed twice with 0.1 M NaH_2_PO_4_ for 30 min and successively dehydrated in 50% ethanol (EtOH), 75% EtOH, 95% EtOH (30 min each), and finally in 100% EtOH (twice for 30 min each time). Propylene oxide (two 15-min washes) was used as a transitional solvent. Cells then were preinfiltrated overnight at a 50:50 ratio propylene oxide to resin (composed of 450 ml dodecenyl succinic anhydride, 250 ml araldite 6005, 82.5 ml Epon 812, 12.5 ml dibutyl phthalate, and 450 μl benzyldimethylamine). The following day, cells were infiltrated with 100% resin for 5 h and subsequently embedded in fresh resin. The embedded cells were sectioned with an ultramicrotome at a thickness of 90 nm and collected on copper mesh grids. The sections were stained with 4% aqueous uranyl acetate for 30 min and for 2 min in 0.2% lead citrate in 0.1 N NaOH. Finally, samples were washed and allowed to dry before imaging again on the FEI Talos 120C transmission electron microscope.

### Statistical analysis.

Results are shown as means ± standard deviations from at least three independent experiments. Data were analyzed using unpaired Student's *t* test or analysis of variance (ANOVA) followed by Tukey’s honestly significant difference test. A *P* value of <0.05 was considered statistically significant.

## Supplementary Material

Supplemental file 1
