# Supplementary material for: Rainbow Kaposi's Sarcoma-Associated Herpesvirus Revealed Heterogenic Replication with Dynamic Gene Expression
Source: J Virol. 2020 Mar 31;94(8):e01565-19. doi: 10.1128/JVI.01565-19 (PMC7108829; doi:10.1128/JVI.01565-19)
Supplement: Supplemental file 1 [file JVI.01565-19-s0001.pdf]

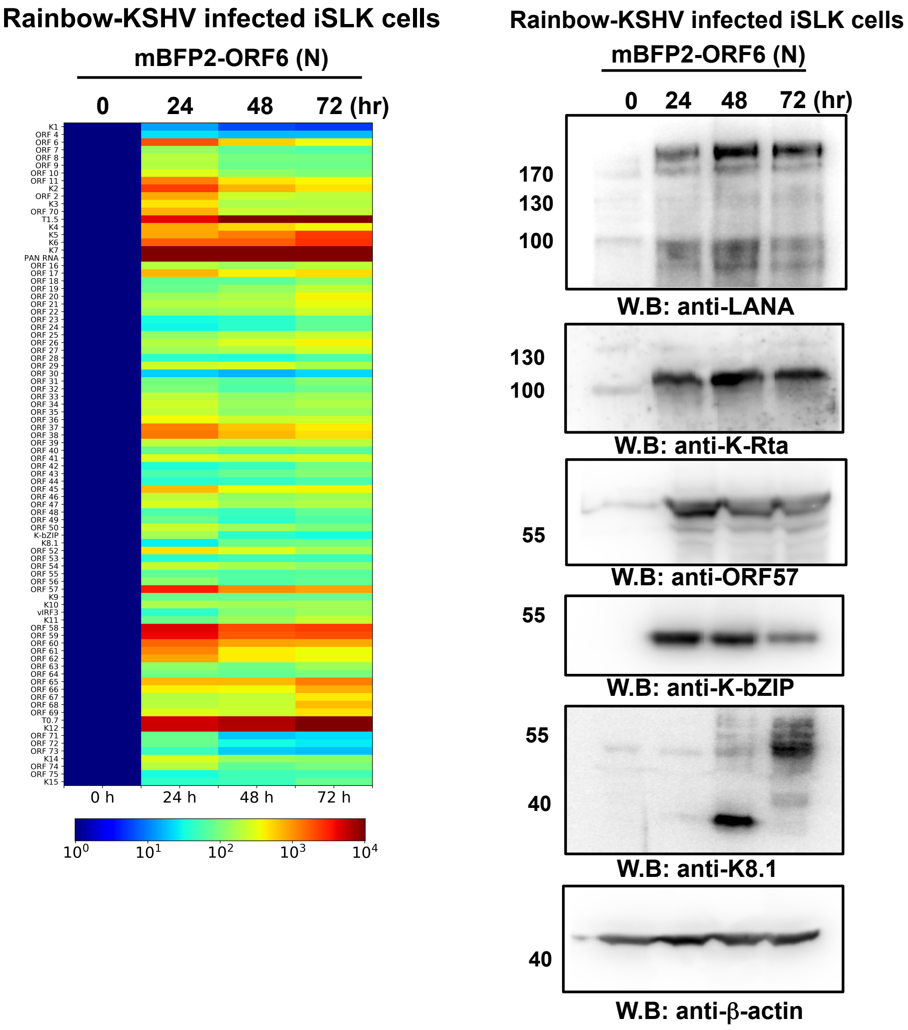

S-Figure 1. (A) Viral gene expression. Rainbow-KSHV infected cells were stimulated with doxycycline and sodium butyrate. Total RNAs were purified at indicated time points and subjected to KSHV PCR arrays. Gene expression is shown as a heatmap. 18S ribosomal RNA was used as an internal standard for normalization and 0 hr time point was set as 1. **(B) Viral protein expression.** Total cell lysates were prepared at indicated time points and subjected to immunoblotting. KSHV proteins and β-actin protein were probed with specific antibodies.

S-Figure 1

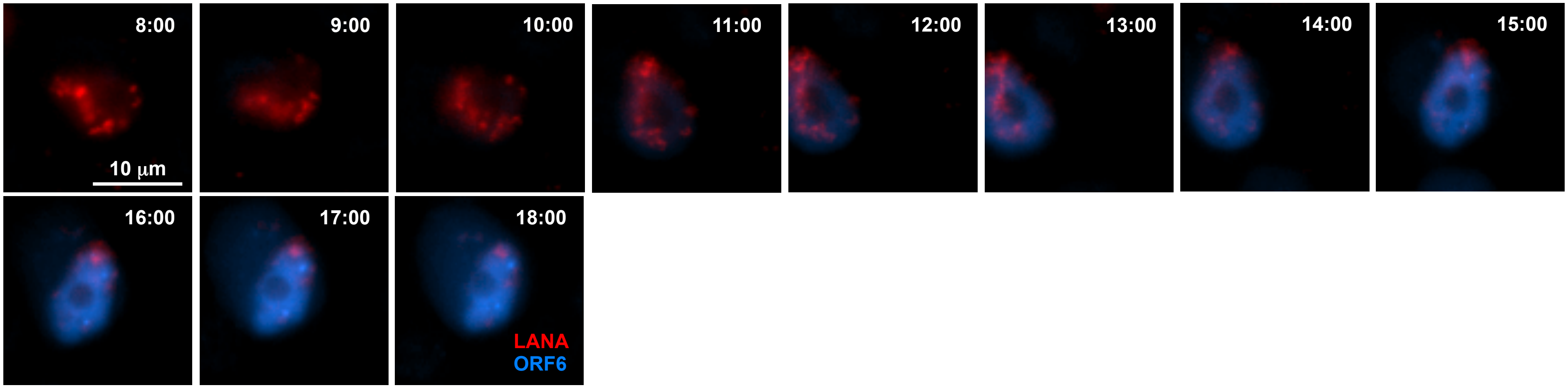

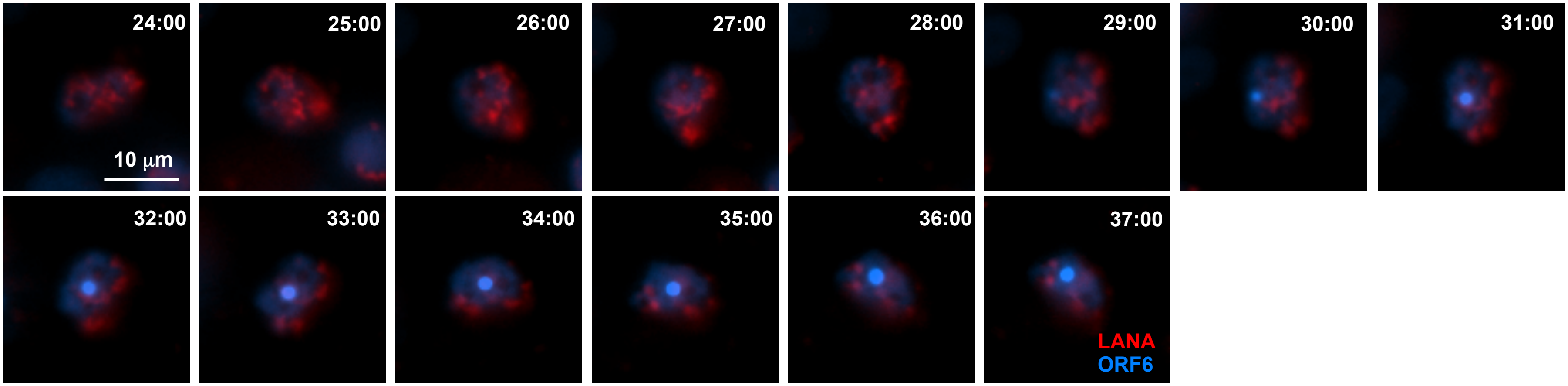

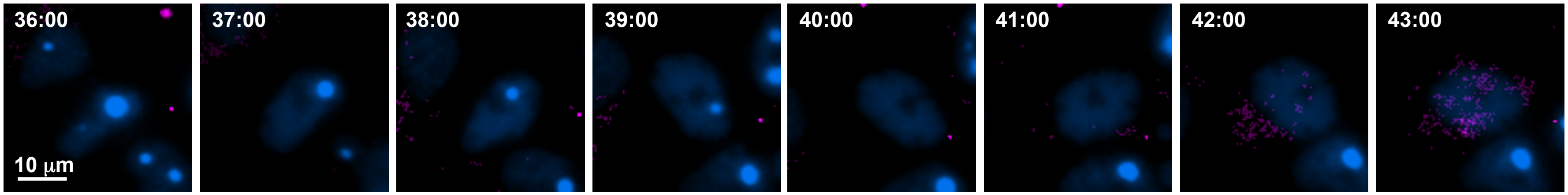

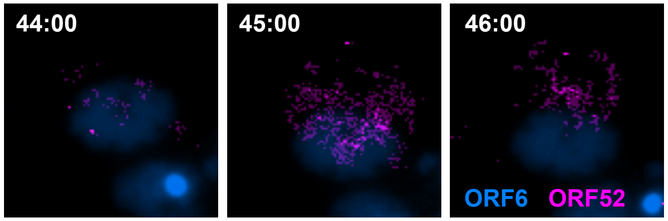

S-Figure 2. Expression dynamics of mCherry-LANA, mBFP-ORF6, and mCardinal-ORF52 during reactivation. iSLK cells latently infected with Rainbow-KSHV were stimulated with doxycycline and TPA. Montages show time lapse of 1-hr interval of each fluorescence proteins. Numbers indicate time (HH:MM) after addition of doxycycline and TPA.

S-Figure 2

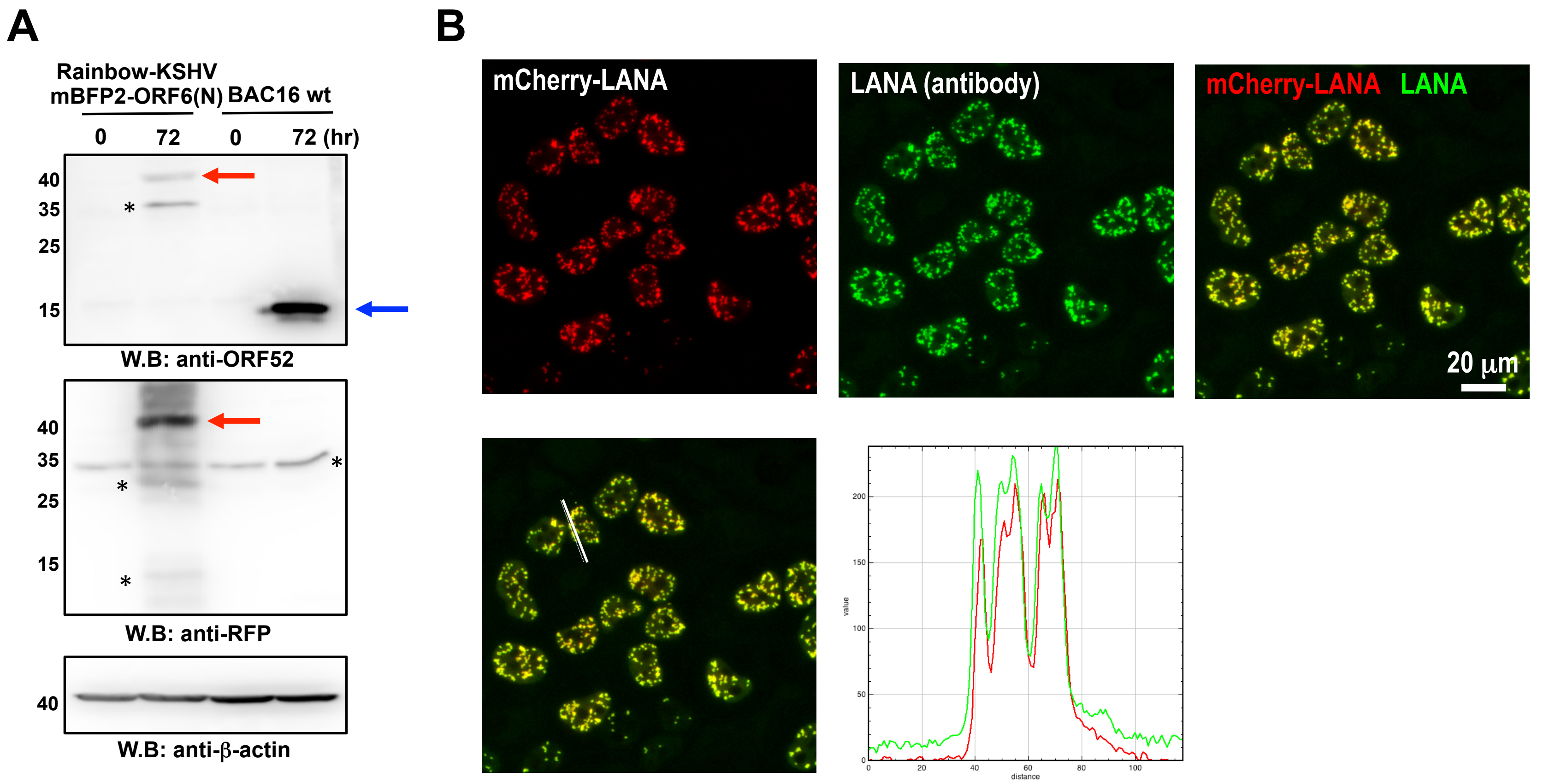

S-Figure 3. Fluorescence fusion protein expression. (A) Expression of mCardinal-ORF52 as a fusion protein in stimulated cells. Total cell lysates were prepared from iSLK cells infected with Rainbow-KSHV at 0 and 72 hr after stimulation with doxycycline and TPA, and subjected to immunoblotting. mCardinal-ORF52 was visualized with anti-ORF52 antibody. Red and blue arrows indicate mCardinal-ORF52 protein (approx. 41 kDa) and ORF52 protein, respectively. Asterisks represent degradation products or nonspecific protein bands. **(B) Distribution of mCherry-LANA fluorescence correlates with anti-LANA antibody staining pattern.** iSLK cells infected with Rainbow-KSHV were fixed, and LANA protein was stained with anti-LANA antibody followed by Alexa 647-conjugated secondary antibody. Bottom panels show line profile of the dots. Green and red line represents signal derived from antibody staining and mCherry, respectively.

S-Figure 3

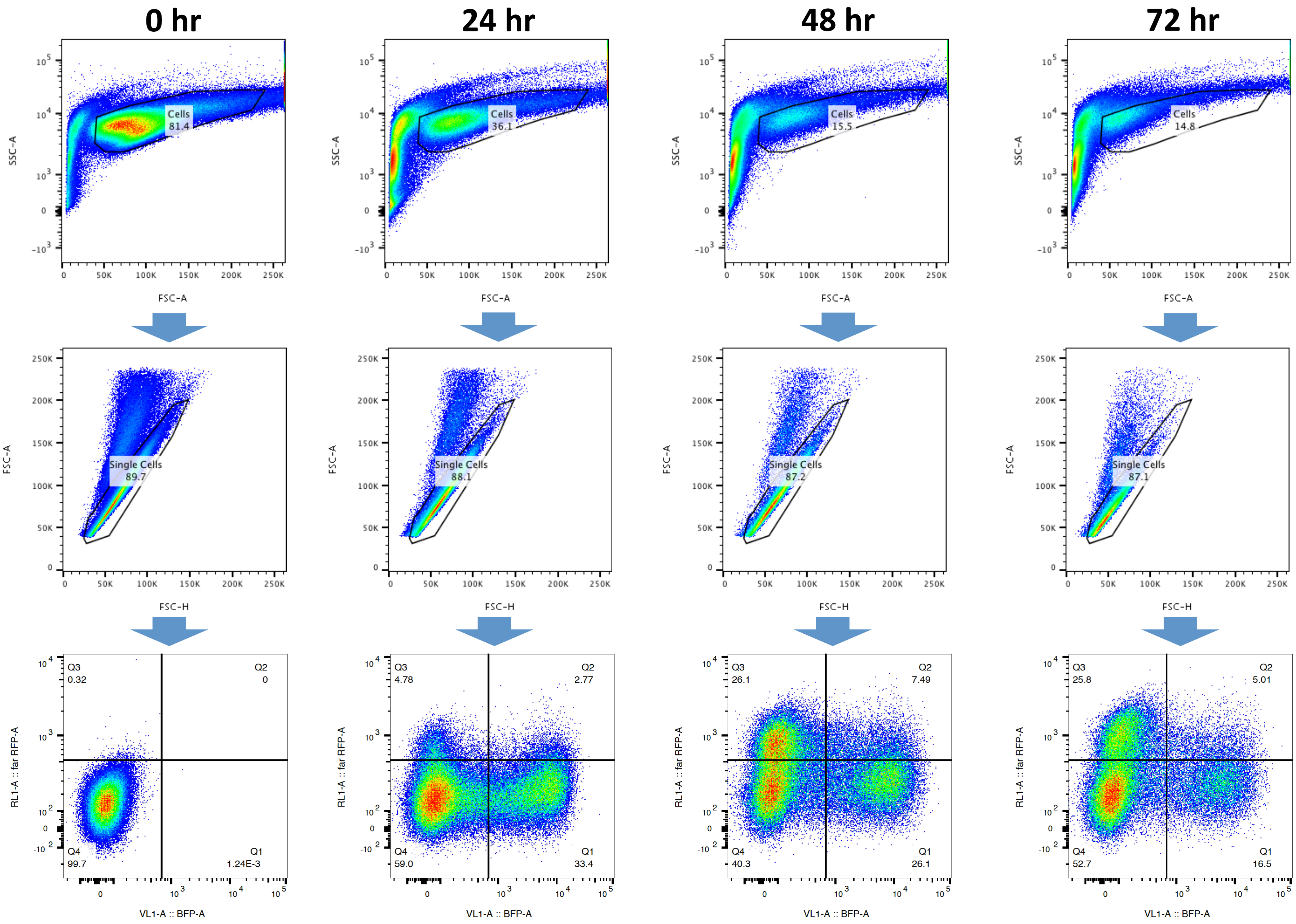

S-Figure 4. Gating positions for FACS analyses presented in Figure 4A. Gating positions described in Figure 4 are shown. Bottom panels are presented in Figure 4A.

S-Figure 4

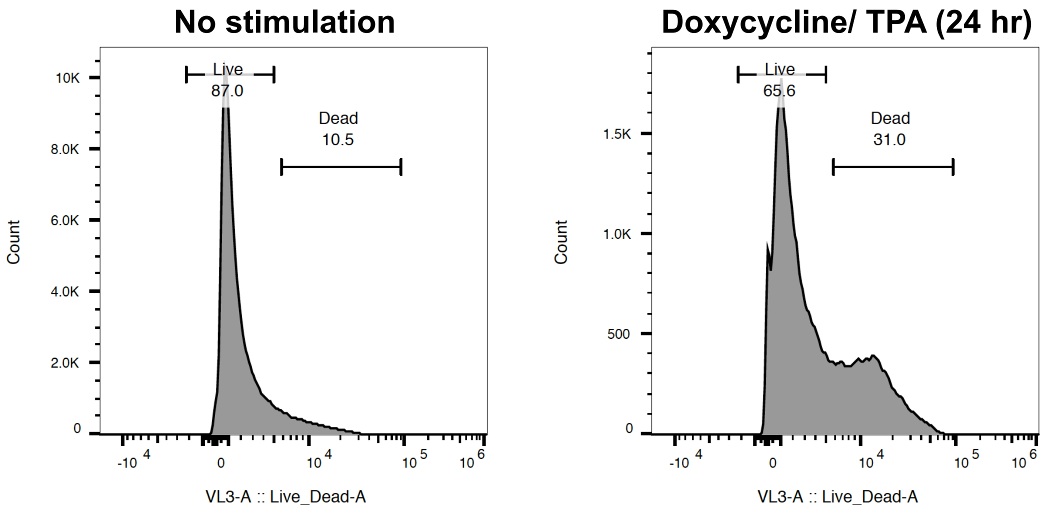

S-Figure 5. Live/Dead staining of stimulated cells. iSLK cells infected with Rainbow-KSHV were stimulated with doxycycline and TPA for 24 hr. The cells were stained with LIVE/DEAD™ Fixable Yellow Dead Cell Stain Kit and analyzed by flow cytometry.

S-Figure 5

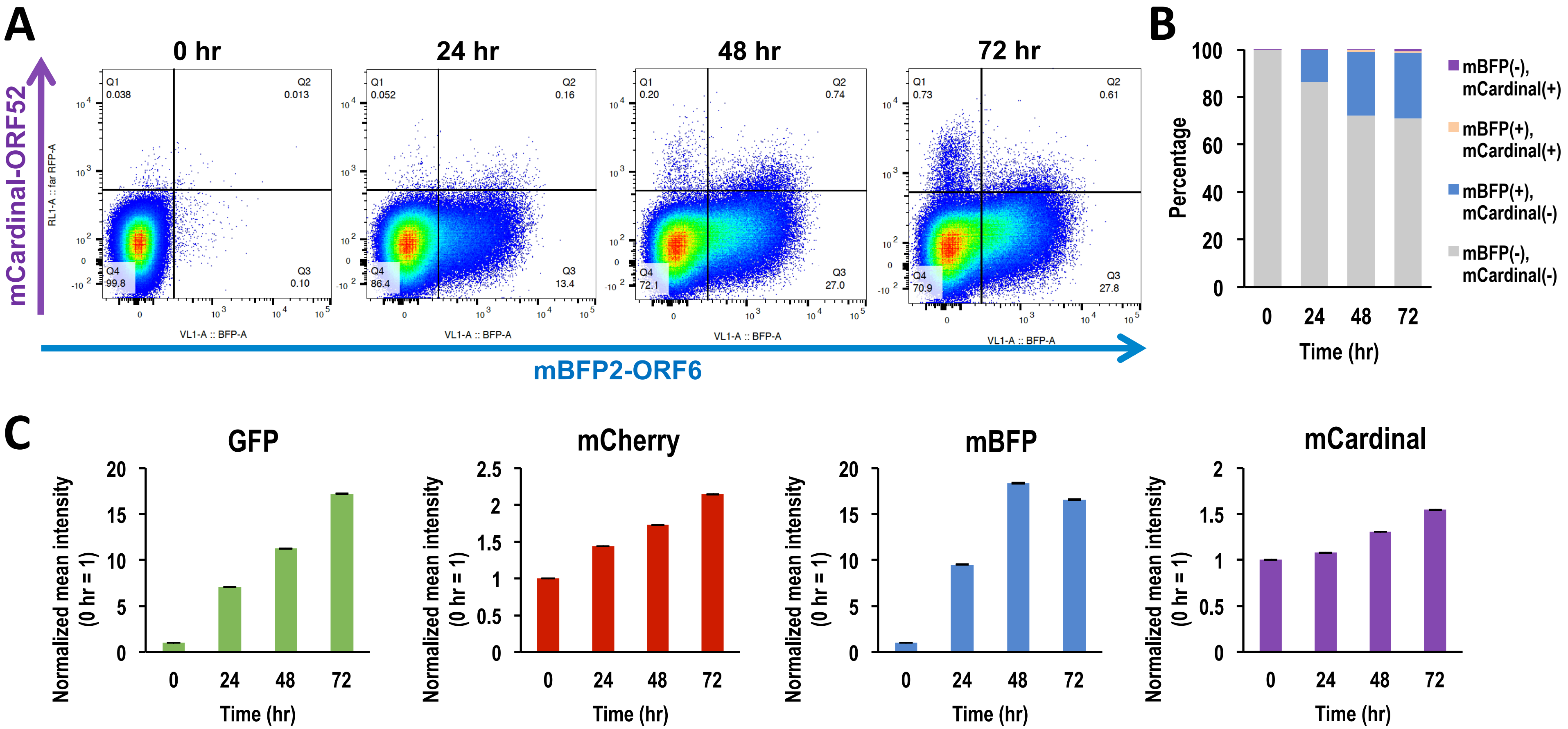

S-Figure 6. Flow cytometry analysis of Rainbow-KSHV infected 293FT cells. (A) Profile of mBFP-ORF6 and mCardinal-ORF52 expression after stimulation. 293FT cells infected with Rainbow-KSHV were stimulated with TPA and sodium butyrate. The cells were fixed with 2% paraformaldehyde at indicated time point, and expression of GFP, mCherry-LANA, mBFP-ORF6, and mCardinal-ORF52 was analyzed by flow cytometry.

(B) Combination of fluorescence. Percentage of cell populations with fluorescence positive and negative cells are shown in bar chart.

(C) Mean intensities of each fluorescence proteins. Changes of mean fluorescence intensities were calculated. Fluorescence intensity at 0 h was set as 1.

S-Figure 6

Doxycycline /TPA	Bortezomib (4 nM)	Chloroquine (50 μM)	BFP (-) mCardinal (-)	BFP (+) mCardinal (-)	BFP (+) mCardinal (+)	BFP (-) mCardinal (+)
-	-	-	100	0	0	0
-	+	-	97.1	2.62	0.13	0.17
-	-	+	94.6	3.99	0.61	0.76
+	-	-	84.3	7.18	1.93	6.56
+	+	-	84.6	6.73	1.82	6.85
+	-	+	82.5	7.22	2.07	8.18

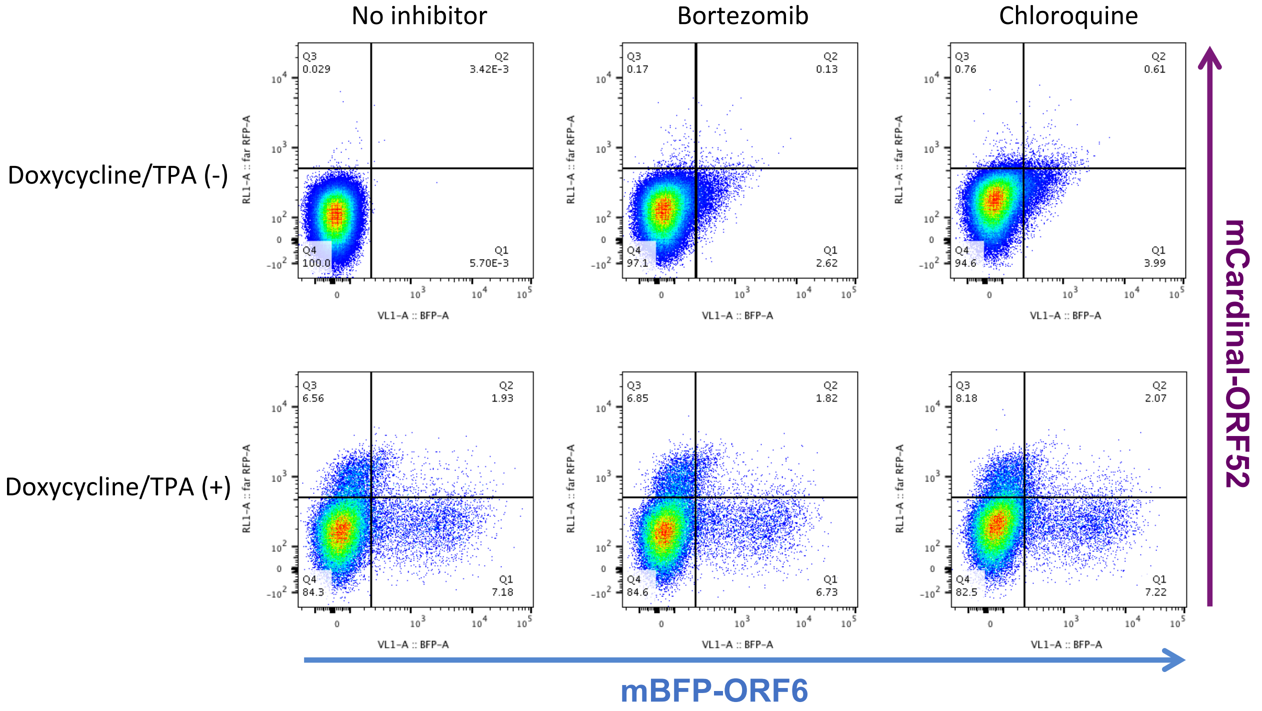

S-Figure 7. Proteasome or lysosome inhibitor did not rescue mBFP2 expression in mCardinal positive cells: iSLK cells infected with Rainbow-KSHV was stimulated with doxycycline and TPA for 6 hr. At 24 hr after stimulation, Bortezomib (4 nM) or chloroquine (50 μM) was added in culture media and incubated for another 24 hr. Cells were fixed with paraformaldehyde. Expression of mBFP-ORF6 and mCardinal-ORF52 was analyzed by flow cytometry, as similar to Figure 5A.

S-Figure 7

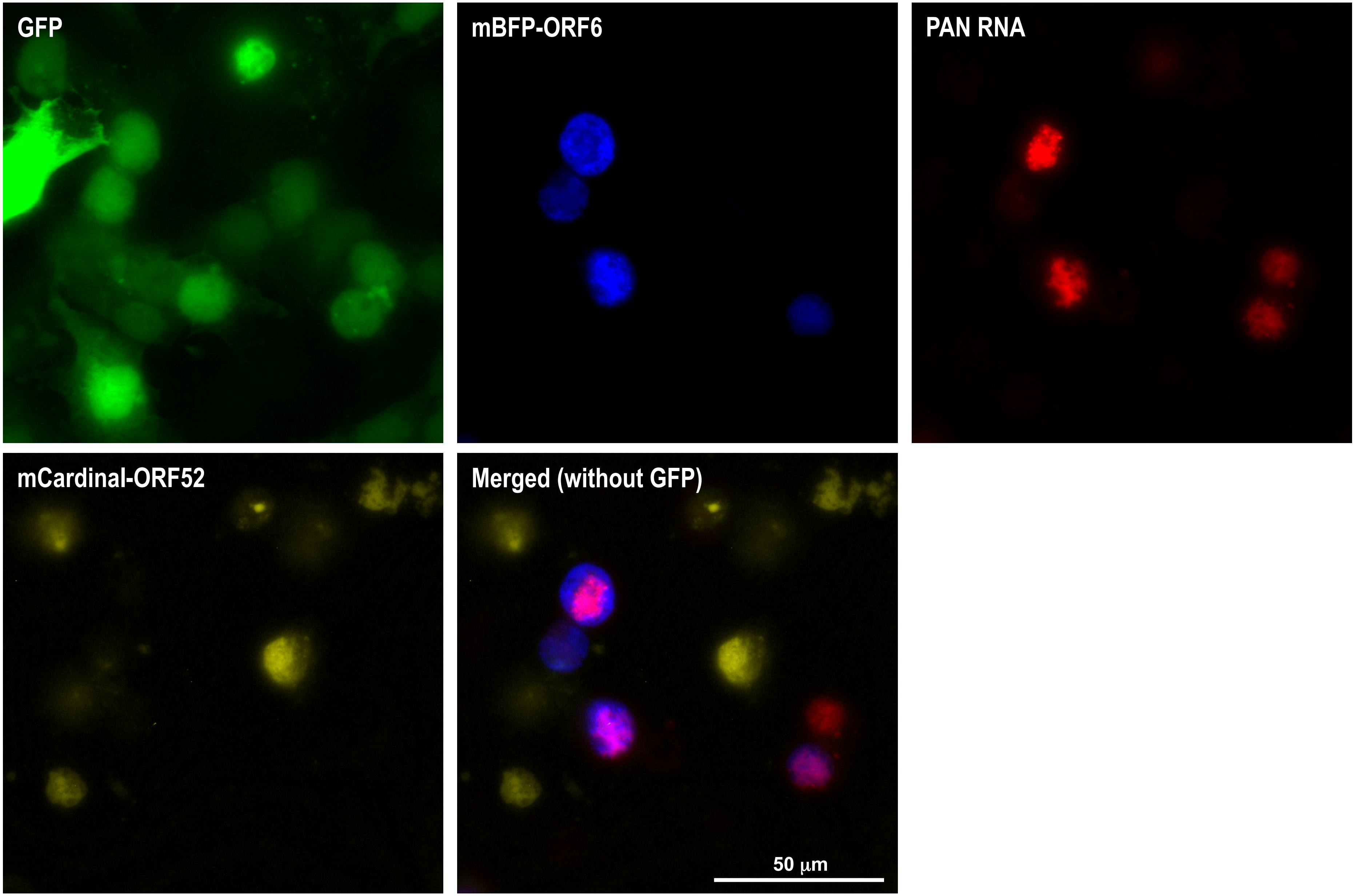

S-Figure 8. Down regulation of PAN RNA in mCardinal-ORF52 expressing cells. PAN RNA was visualized by hybridizing tiling oligo probes labeled with Quasar 570. Individual panels for mBFP2-ORF6 (blue), mCardinal-ORF52 (yellow), PAN RNA (red) are shown.

S-Figure 8
